# Resistance to First-Line Anti-TB Drugs Is Associated with Reduced Nitric Oxide Susceptibility in *Mycobacterium tuberculosis*

**DOI:** 10.1371/journal.pone.0039891

**Published:** 2012-06-29

**Authors:** Jonna Idh, Mekidim Mekonnen, Ebba Abate, Wassihun Wedajo, Jim Werngren, Kristian Ängeby, Maria Lerm, Daniel Elias, Tommy Sundqvist, Abraham Aseffa, Olle Stendahl, Thomas Schön

**Affiliations:** 1 Division of Microbiology and Molecular Medicine, Faculty of Health Sciences, Linköping University, Linköping, Sweden; 2 Department of Microbiology, Immunology and Parasitology, Addis Ababa University, Debre Zeit, Ethiopia; 3 Armauer Hansen Research Institute, Addis Ababa, Ethiopia; 4 Department of Microbiology and Parasitology, Gondar University Hospital, Gondar, Ethiopia; 5 Unit of Highly Pathogenic Microorganisms, Department of Preparedness, Swedish Institute for Communicable Disease Control, Solna, Sweden; 6 Clinical Microbiology, MTC, Karolinska Institute, Karolinska University Hospital, Stockholm, Sweden; 7 Department of Cancer and Inflammation, Institute of Molecular Medicine, University of Southern Denmark, Odense, Denmark; 8 Department of Infectious Diseases and Department of Clinical Microbiology, Kalmar County Hospital, Kalmar, Sweden; Statens Serum Institute, Denmark

## Abstract

**Background and Objective:**

The relative contribution of nitric oxide (NO) to the killing of *Mycobacterium tuberculosis* in human tuberculosis (TB) is controversial, although this has been firmly established in rodents. Studies have demonstrated that clinical strains of *M. tuberculosis* differ in susceptibility to NO, but how this correlates to drug susceptibility and clinical outcome is not known.

**Methods:**

In this study, 50 sputum smear- and culture-positive patients with pulmonary TB in Gondar, Ethiopia were included. Clinical parameters were recorded and drug susceptibility profile and spoligotyping patterns were investigated. NO susceptibility was studied by exposing the strains to the NO donor DETA/NO.

**Results:**

Clinical isolates of *M. tuberculosis* showed a dose- and time-dependent response when exposed to NO. The most frequent spoligotypes found were CAS1-Delhi and T3_ETH in a total of nine known spoligotypes and four orphan patterns. There was a significant association between reduced susceptibility to NO (>10% survival after exposure to 1 mM DETA/NO) and resistance against first-line anti-TB drugs, in particular isoniazid (INH). Patients infected with strains of *M. tuberculosis* with reduced susceptibility to NO showed no difference in cure rate or other clinical parameters but a tendency towards lower rate of weight gain after two months of treatment, independent of antibiotic resistance.

**Conclusion:**

There is a correlation between resistance to first-line anti-TB drugs and reduced NO susceptibility in clinical strains of *M. tuberculosis*. Further studies including the mechanisms of reduced NO susceptibility are warranted and could identify targets for new therapeutic interventions.

## Introduction

An important part of the host defence against *Mycobacterium tuberculosis* is nitric oxide (NO) produced by activated macrophages from L-arginine, molecular oxygen, and NADPH through the activity of inducible NO synthase (iNOS) [1]. Several animal and macrophage experiments have shown that NO and related reactive nitrogen species (RNS) constitute a major host defence mechanism against intracellular pathogens including, *M. tuberculosis* in both the acute and the latent phases of infection [2], [3]. It has also been shown that RNS are actively produced in human tuberculosis (TB), although their relative importance is controversial [4]–[9]. We have previously found that patients with TB have lower levels of NO in exhaled air and lower levels of NO metabolites in urine, compared to healthy controls [10], and that nutritional supplements of L-arginine can mediate clinical improvement in HIV-negative TB patients [11], [12].

Bicyclic nitroimidazoles are drug candidates currently in phase II trials for the treatment of tuberculosis [13], [14]. They are prodrugs, and one of the active metabolites generates RNS, which has been suggested to mediate the major anaerobic effect of the drug [15], [16]. It has also been shown that NO can contribute to the antimycobacterial action of the prodrug isoniazid (INH) [17]. The exact mechanisms of INH action are not fully understood, but during activation by mycobacterial catalase-peroxidase (encoded by the *katG* gen), several INH-derived intermediates and free radicals have been suggested to be of importance [17], [18].

Since previous studies have shown that susceptibility of mycobacteria to RNS can be strain-dependent [19]–[24], we here investigated the impact of relative NO susceptibility of *M. tuberculosis* on the clinical course of TB and in relation to drug resistance.

## Materials and Methods

### Patients

Patients presented here have previously been described in a clinical trial on arginine-rich food supplementation (ClinicalTrials.gov identifier: NCT00857402) were smear-positive patients with pulmonary TB, given Directly Observed Treatment Short course (DOTS) at Gondar University Hospital, Ethiopia, were consecutively asked to participate in the study. The inclusion criteria were age 15 to 60 years, willingness to take part in the study and no known chronic disease except TB and HIV. Baseline data for age, sex, HIV serostatus, presence of BCG scar, body mass index (BMI), body temperature, cough and haemoptysis was registered. Chest X-ray findings were graded as minimal, moderate or far advanced TB [25], and after two months of treatment as normal, marked regression, regression, stable disease or progression. Laboratory parameters, erythrocyte sedimentation rate (ESR), baseline acid-fast bacilli (AFB) smear grade, and NO metabolites in urine (as described by Verdon et al [26]) were measured. In the first eleven included subjects, exhaled NO was registered using an chemiluminescence NO analyser (NIOX, Aerocrine AB, Sweden) as previously described [27].

### Identification and Spoligotyping of *M. tuberculosis*

One sputum specimen from each patient was kept at −20°C (as presented before) [28]. The sputum was decontaminated (modified Petroff’s method) and processed according to standard protocols [29] with subsequent inoculation on Lowenstein Jensen (LJ) slant media. *M. tuberculosis* was identified by a PCR-based approach built on genomic deletion analysis [30], and was carried out as described [31]. Spoligotyping was performed using a standard protocol [32] with the oligonucleotides DRa and DRb to amplify the direct repeat (DR) regions.

### Drug Susceptibility Testing

Drug susceptibility testing for streptomycin (SM, 2 mg/L), isoniazid (INH, 1 mg/L), ethambutol (EMB, 5 mg/L) and rifampin (RIF, 1 mg/L) was performed using the indirect proportion method on LJ media, as recommended by WHO [33], [34]. When bacterial growth on the antibiotic-free media reached 50–100 colonies, a growth more than 1% on antibiotic-containing media was classified as resistant.

### Susceptibility Testing for Nitric Oxide

*M. tuberculosis* from LJ medium was cultured in Middlebrook 7H9 broth supplemented with oleic acid-albumin-dextrose-catalase (OADC) until log phase (determined by bacterial growth as colony forming units (CFU) at different incubation periods, data not shown). Following washing in sterile PBS/0.05% Tween 80, a final concentration of 10^7^ CFU/mL of bacteria were exposed in duplicates to 1 mM diethylenetriamine/nitric oxide adduct (DETA/NO, Sigma-Aldrich) and PBS (control) separately in Middlebrook 7H9 medium without OADC for 24 hours at 37°C. *M. tuberculosis* H37Rv (H37Rv) and *M. bovis* BCG (BCG) were used as reference strains in dose-response studies. The antimicrobial effect of DETA/NO was determined by viable count (CFUs) in tenfold dilutions on Middlebrook 7H10 plates supplemented with glycerol and 10% OADC.

### Ethical Considerations

This study was conducted according to the principles expressed in the Declaration of Helsinki and the study was approved by the ethics committees at the Research and Publication Office at Gondar College of Medicine and Health Sciences, Ethiopian Science and Technology Commission, and Karolinska Hospital, Sweden. All study subjects were included only after obtaining written informed consent. For children below 18 years informed and written consent was obtained from parents or guardians.

### Statistical Analyses

Data are presented as median and rang or first and third quartiles (Q1–Q3) if not stated otherwise. Numerical data were analysed with Mann-Whitney *U*-test, discrete data with Pearson’s Chi-square test and Fisher’s exact test, and *p*-values of less than 0.05 were considered as statistically significant. Multiple logistic regression analysis with a stepwise correction was applied on variables with a *p*-value less than 0.1 in the univariate analysis.

## Results

### Baseline Characteristics

A total of 50 patients with confirmed culture- and smear-positive pulmonary TB were included in the study. The median age was 27 years (range 15–59), 50% (25/50) were female and 44% (22/50) were HIV-positive. Characteristics are presented in detail in table 1.

**Table 1:** Characteristics for patients infected with strains of *M. tuberculosis* susceptible to NO or with reduced susceptibility to NO.

		NO-susceptible		Reduced NO susceptibility		
		N	Median (Q1–Q3)	N	Median (Q1–Q3)	*p*
Characteristics	Age (years)	26	26.0 (20.8–37.0)	24	27.0 (23.2–37.0)	NS
	BMI (kg/m^2^)	26	16.8 (15.6–18.0)	24	15.8 (14.7–17.4)	NS
	Body temperature (°C)	25	37.6 (36.5–38.9)	24	37.8 (37.1–38.7)	NS
	ESR week 0 (mm/h)	26	72.5 (50.0–85.5)	24	78.0 (67.5–88.5)	NS
	ESR week 8 (mm/h)	25	44.0 (12.0–78.0)	24	41.0 (27.5–62.0)	NS
		N	% (*n*)	N	% (*n*)	*p*
	Females	26	46.2 (12)	24	54.2 (13)	NS
	HIV	26	38.5 (10)	24	50.0 (12)	NS
	BCG	26	11.5 (3)	24	12.5 (3)	NS
	Cough week 0	26	100.0 (26)	24	100.0 (24)	NS
	Cough week 8	25	64.0 (16)	24	29.2 (7)	<0.05
	Haemoptysis week 0	26	23.1 (6)	24	20.8 (5)	NS
	Haemoptysis week 8	25	8.0 (2)	24	4.2 (1)	NS
	Weight gain from week 0 to 8	25	100.0 (25)	24	83.3 (20)	0.05
	Sputum conversion week 8	26	84.6 (22)	24	83.3 (20)	NS
Chest X-ray week 0	Normal	19	0.0 (0)	16	0.0 (0)	NS
	Minimal	19	26.3 (14)	16	25.0 (4)	NS
	Moderately advanced	19	52.6 (10)	16	50.0 (8)	NS
	Far advanced	19	21.1 (4)	16	25.0 (4)	NS
Chest X-ray week 8	Normal	16	0.0 (0)	15	6.7 (1)	NS
	Marked regression	16	6.3 (1)	15	13.3 (2)	NS
	Regression	16	62.5 (10)	15	66.7 (10)	NS
	No change	16	25.0 (4)	15	13.3 (2)	NS
	Progress	16	6.3 (1)	15	0.0 (0)	NS
Outcome	Cured	26	76.9 (20)	24	70.8 (17)	NS
	Died	26	3.8 (1)	24	0.0 (0)	NS
	Treatment failure	26	11.5 (3)	24	8.3 (2)	NS
	Defaulter	26	3.8 (1)	24	12.5 (3)	NS
	Transferred out	26	3.8 (1)	24	4.2 (1)	NS
Antibiotic resistance	Fully susceptible	26	96.2 (25)	22	66.7 (16)	<0.05
	INH resistance	26	0.0 (0)	24	20.8 (5)	<0.05
	SM resistance	26	3.8 (1)	24	8.3 (2)	NS
	EMB resistance	26	0.0 (0)	24	0.0 (0)	NS
	RIF resistance	26	0.0 (0)	24	4.2 (1)	NS
		N	Median (Q1–Q3)	N	Median (Q1–Q3)	*p*
NO production	Urinary NO week 0 (mM)	26	1111 (792–1615)	24	1264 (931–1810)	NS
	Urinary NO week 8 (mM)	25	1067 (961–1976)	24	1436 (881–2276)	NS
	Exhaled NO week 0 (ppb)	11	15.8 (14.2–20.2)	8	12.4 (8.1–15.2)	NS
	Exhaled NO week 8 (ppb)	11	15.7 (11.3–19.5)	8	15.0 (11.1–24.1)	NS

Q1–Q3 (quartile 1 to quartile 3); NO (nitric oxide); BMI (body mass index); ESR (sedimentation rate); INH (isoniazid); SM (streptomycin); EMB (ethambutol); RIF (rifampin). NO-susceptible and reduced NO-susceptible strains defined as ≤10% and >10% survival respectively after exposure to 1 mM DETA/NO. All patients were smear positive at week 0. Continuous data were tested with Mann-Whitney *U*-test and discrete data with Fisher’s exact test or Pearson’s Chi-square test.

### Dose- and Time-dependent Response to NO

**Figure 1:**
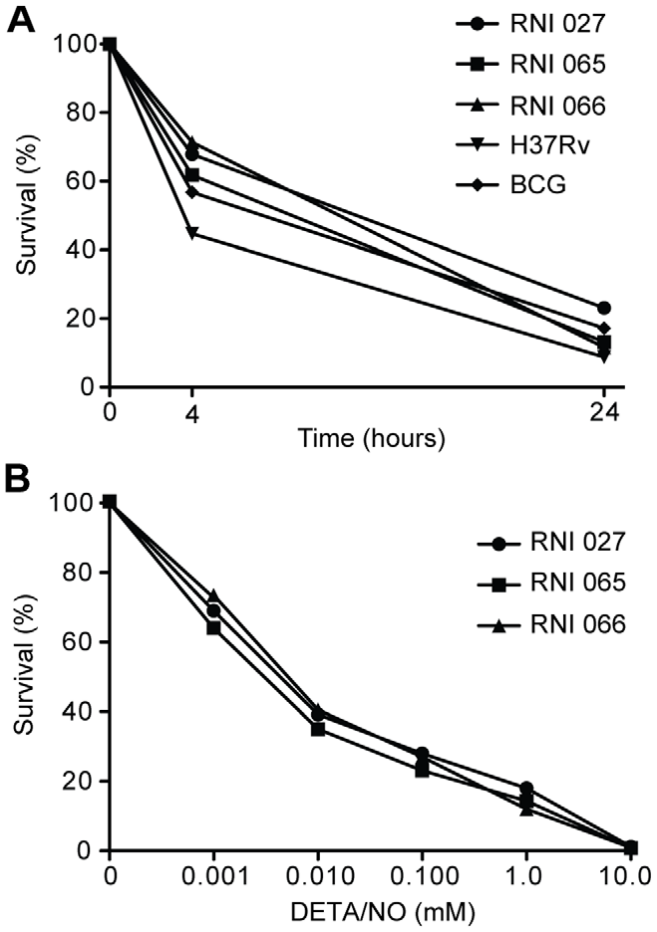
Dose- and time-dependent killing of *M. tuberculosis* exposed to NO. Survival of three clinical strains, H37Rv and BCG after exposure to 1 mM of the NO donor DETA/NO for 4 and 24 hours (A). Survival of the three clinical strains exposed to different doses of DETA/NO for 24 hours (B). Survival was determined through viable count (colony forming units, CFU) and each point represents a mean value of duplicates.

In order to determine doses and time points for the NO exposure, initial experiments with three randomly selected clinical isolates, one resistant to INH (coded RNI 027) and two fully susceptible to first-line anti-TB drugs (RNI 065 and RNI 066), were performed in duplicates. There was a dose- and time-dependent killing when exposed to DETA/NO (0.1–10 mM) (figure 1). After a 4-hour exposure to the selected dose of 1 mM DETA/NO, the mean survival rate of the strains for the clinical isolates was 68% (RNI 027), 62% (RNI 065), and 71% (RNI 066). In H37Rv and BCG, the survival rate was 45% and 57% respectively. The response was time-dependent and 24 hours post-exposure, the survival decreased to 23% (RNI 027), 13% (RNI 065) and 12% (RNI 066) for the clinical isolates and to 9% and 17% for H37Rv and BCG, respectively (figure 1b).

### Association between Reduced Susceptibility to NO and Resistance to First-line anti-TB Drugs

Drug susceptibility testing showed an overall resistance rate to first-line anti-TB drugs of 18% (9/50). The most frequent resistance was found against INH (10%, 5/50) followed by SM (6%, 3/50) and RIF (2%, 1/50). No strains resistant to EMB and no multidrug-resistant (MDR) TB strains were isolated in this study. The ratio of resistant strains did not differ between HIV-positive and HIV-negative individuals (14% (3/22) vs. 21% (6/28), *p* = 0.713).

**Figure 2:**
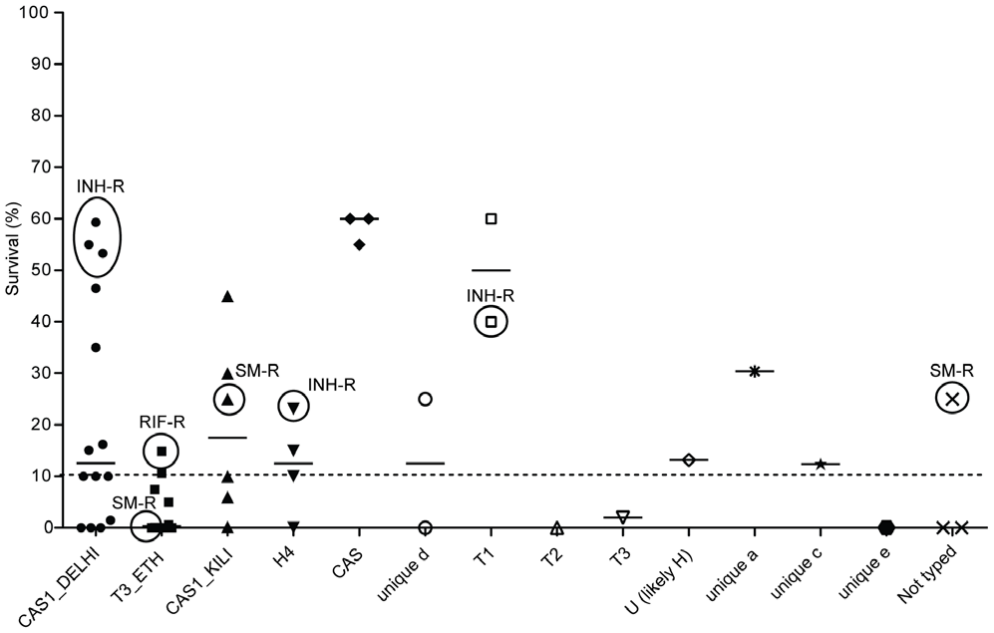
Reduced NO susceptibility in spoligotype-based clusters of *M. tuberculosis*. Survival of clinical isolates 24 hours after exposure to the NO donor DETA/NO. Presence of resistance to first-line anti-TB drugs is indicated with circles; isoniazid (INH), streptomycin (SM) and rifampin (RIF). Each point represents a mean value of duplicates and the dashed line is the median survival of all 50 isolates.

The median survival in the 50 clinical isolates, 24 hours post exposure to 1 mM DETA/NO, was 10% and showed a variation between 0 to 60% when compared to a control exposed to PBS (p<0.001, figure 2). For further comparison of susceptibility to NO with drug resistance and clinical outcome, reduced susceptibility to DETA/NO was defined as >10% survival (1 log_10_) after exposure to 1 mM DETA/NO for 24 hours compared to the control exposed to PBS.

Strikingly, no INH resistance was detected among NO-susceptible strains and the median survival of INH-resistant isolates exposed to DETA/NO was 53% (Q1–Q3, 32–57%, n = 5) compared to 10% (Q1–Q3, 0–25%, n = 45, *p* = 0.006) in INH-susceptible isolates.

### Clinical Response in Relation to NO Susceptibility of *M. tuberculosis*

Infection with strains of *M. tuberculosis* with reduced susceptibility to NO did not correlate to the final clinical outcome according to WHO (cured, died, treatment failure, defaulter or transferred out; table 1), but there was a tendency to a lower rate of weight gain after 8 weeks of treatment (83% (20/24) vs. 100% (25/25), *p* = 0.05). Three out of the four study subjects who did not increase in weight during the 8 first weeks of treatment were HIV-positive, but there was no statistical difference in weight gain overall between HIV-positive and HIV-negative TB patients (86% (18/21) vs. 96% (27/28), *p* = 0.301). Nor did patients infected with drug-resistant strains differ in their rate of weight gain compared to patients with drug-susceptible strains (8/9, 89% vs. 37/40, 93%, *p* = 0.569).

### Multiple Logistic Regression Analysis

The multiple logistic regression analysis showed that reduced NO susceptibility was associated to drug resistance also when age, sex, HIV status and supplementation of arginine-rich food were included in in the analysis (OR 19.00; 95% CI (1.67–215.52), *p* = 0.019, n = 50, table 2).

**Table 2:** Multiple logistic regression analysis comparing strains of *M. tuberculosis* susceptible to NO with strains of reduced susceptibility to NO.

			Reduced NO susceptibility			Univariate			Multivariate	
Parameter		N	n	%	OR (95% conf. int.)		*p*	OR (95% conf. int.)		*p*
Age	≤28	30	13	43.4	1.00			1.00		
	>28	20	11	55.0	1.60 (0.50–5.15)		0.424	2.34 (0.50–10.96)		0.275
Gender	M	25	11	44.0	1.00			1.00		
	F	25	13	52.0	1.38 (0.44–4.32)		0.574	2.55 (0.63–10.25)		0.182
HIV	No	28	12	42.9	1.00			1.00		
	Yes	22	12	54.5	1.60 (0.50–5.07)		0.417	1.72 (0.41–7.27)		0.454
Arginine supplementation	No	24	14	58.3	1.00			1.00		
	Yes	26	10	38.5	0.45 (0.14–1.43)		0.169	0.38 (0.09–1.53)		0.168
Resistance to first- lineanti-TB drugs	No	41	16	39.0	1.00			1.00		
	Yes	9	8	88.9	12.50 (1.34–116.53)		0.027	19.00 (1.67–215.52)		0.019

### Spoligotype Patterns

Of the 50 clinical strains 47 (94%) were successfully typed by conventional spoligotyping technique [32]. The isolates were identified as five orphan spoligotypes and nine spoligotypes present in the *M. tuberculosis* molecular markers database (SITVIT) and compared to the fourth international spoligotyping database (SpolDB4) for classification [35]–[37]. The most common spoligotypes were CAS1-Delhi (28%, 13/47) and T3_ETH (23%, 11/47). Although the spoligotype-based clusters differed in susceptibility to NO, our sample size was not sufficient to investigate any significant differences between the clusters (figure 2).

## Discussion

The main finding of this study is that reduced susceptibility to NO in clinical strains of *M. tuberculosis* was associated with resistance to first-line drugs against TB. As previously reported, certain strains of *M. tuberculosis* can to some extent resist RNS generated *in vitro* as acidified nitrite (*M. intracellulare* 31F093T, KUMS 9007 [19], *M. tuberculosis* CDC1551, CB3.3 [23], *M. bovis*, *M. tuberculosis* 79499 [21], a C strain cluster defined by IS6110-based strain-typing [20], and the genotypes G1, G2, S2, U [24]). Since the acid has a bactericidal effect in itself and actually potentiates the effect of NO [38], it has been acknowledged that the new generation NO donors (DETA/NO) can better mimic a prolonged release with low levels of NO similar to the production *in vivo* [39]. In dose-response experiments for three clinical strains, we found that there was a dose-dependent killing by one log_10_ at 1 mM DETA/NO and by 2 log_10_ for 10 mM DETA/NO. The level of inhibition by NO at 24 hours was between 0.5 to 2 log_10_ for 1 mM DETA/NO, which could be compared to the early effect of bactericidal drugs such as INH [40]. To discriminate difference in susceptibility to NO between clinical strains, we used a cut-off for reduced NO susceptibility at 10% survival after 24 hours compared to control, which is similar to the wild type distribution used for defining critical concentrations for *M. tuberculosis* [33]. Lower doses of DETA/NO have been used to induce expression of latency-associated genes such as *dosR* [41], [42]. The effect of NO is likely to be increased if investigated at later time points as for anti-TB drugs, since killing of *M. tuberculosis* takes more time relative to other bacteria with higher replication rates such as *E. coli*. The analysis was blinded to antibiotic resistance data, and a high level of antibiotic resistance was observed in the isolates with reduced NO susceptibility. Strikingly, no INH resistance was detected among NO-susceptible strains but on the other hand reduced NO susceptibility was not exclusively found among INH-resistant strains. INH is a prodrug that needs activation by the *katG*-encoded mycobacterial catalase-peroxidase, where several INH-derived intermediates including free radicals have been suggested to contribute to the overall antimycobacterial action of INH [17], [18]. We did not verify the genetic location of the INH resistance, although the *katG*-mutation is the predominant cause of INH resistance in strains of *M. tuberculosis* from Ethiopia [43]. It has previously been shown that the loss of *katG* could render the bacteria susceptible to reactive oxygen species (ROS) and RNS, and a compensatory up-regulation of the alkyl hydroperoxidase (*ahpC*) gene could be the explanation for the increased resistance to NO as a compensatory survival strategy of INH-resistant bacteria [44]–[46]. Other candidates for antioxidant systems up-regulated by *M. tuberculosis* during oxidative stress are thioredoxin-dependent peroxidases (TPx), resulting in tolerance to peroxides produced by the immune system [42]. Most likely, it is a combination of the expression of genes upon exposure to reactive species, the resistance of the *M. tuberculosis* cell wall itself, and up-regulation of genes to repair damaged proteins and DNA, that result in reduced susceptibility against ROS and RNS in *M. tuberculosis* [42]. The most frequent spoligotypes were CAS1Delhi and T3_ETH and a more accurate identification of genetic relationship among the isolates is likely to better have identified differences among the strains in respect to susceptibility to NO.

Although the small sample size is a limitation, no correlation was found between susceptibility of *M. tuberculosis* to NO and treatment outcome, but there was a tendency towards lower rate of weight gain after 8 weeks of treatment, in subjects infected with *M. tuberculosis* with reduced susceptibility to NO. To draw a more definitive conclusion on whether this result was confounded by the presence of antimycobacterial drug resistance, a larger sample size is needed.

In a situation of increasing MDR and extensively drug-resistant (XDR) TB, new drug targets are needed [47]. NO is produced in activated macrophages during TB [1], [8], [9] and based on the correlation between reduced susceptibility to NO and resistance to first-line anti-TB drugs, the relative importance of oxidative defence mechanisms in *M. tuberculosis* needs to be further investigated.
